# Activated Factor X Induces Endothelial Cell Senescence Through IGFBP-5

**DOI:** 10.1038/srep35580

**Published:** 2016-10-18

**Authors:** Fumihiro Sanada, Yoshiaki Taniyama, Jun Muratsu, Rei Otsu, Masaaki Iwabayashi, Miguel Carracedo, Hiromi Rakugi, Ryuichi Morishita

**Affiliations:** 1 Department of Clinical Gene Therapy, Suita, Osaka 565-0871, Japan; 2 Department of Geriatric and General Medicine, Osaka University Graduate School of Medicine, Suita, Osaka 565-0871, Japan

## Abstract

Uncontrolled coagulation contributes to the pathophysiology of several chronic inflammatory diseases. In these conditions, senescent cells are often observed and is involved in the generation of inflammation. The coincidence of hyper-coagulation, cell senescence, and inflammation suggests the existence of a common underlying mechanism. Recent evidence indicates that activated coagulation factor X (FXa) plays a role in the processes beyond blood coagulation. This non-hematologic function entails the mediation of inflammation and tissue remodeling. We therefore tested the hypothesis that FXa induces cell senescence resulting in tissue inflammation and impaired tissue regeneration. Human umbilical vein endothelial cells were stimulated with FXa for 14 days. The proliferation of cells treated with FXa was significantly smaller, and the fraction of senescence-associated β-galactosidase-positive cells was increased as compared to the control group. RT-qPCR array revealed that FXa increased the expression of IGFBP-5, EGR-1, p53, and p16^INK4a^. Inhibition of FXa by a direct FXa inhibitor, rivaroxaban, or IGFBP-5 by siRNA decreased FXa-induced cell senescence, restoring cell proliferation. Moreover, in an ischemic hind limb mouse model, FXa inhibited neovascularization by endothelial progenitor cell. However, rivaroxaban significantly restored FXa-induced impaired angiogenesis. In summary, FXa induced endothelial cell senescence through IGFBP-5, resulting in impaired angiogenesis.

Anti-coagulation therapy targeting cardiovascular diseases has made great advances in recent years^1^. Treatment strategies directly targeting activated coagulation factor X (FXa) have been established for atrial fibrillation and deep vein thrombosis^2^,^3^. However, clinical and basic evidence indicate that FXa has the various activities beyond blood coagulation^4^,^5^. These non-hematologic functions are mainly mediated by protease-activated receptors, PARs, and enhance tissue inflammation and remodeling^4^,^6^,^7^. Although acute (short-term) inflammation leads to tissue repair, chronic inflammation results in undissolved wound healing, which is often seen in atherosclerotic plaques and heart failure^8^. In these pathological conditions, senescent cells are frequently observed and appear to be involved in the progression of chronic inflammation by releasing inflammatory cytokines, senescence-associated secretory phenotype (SASP)^9^. The coincidence of cell senescence, inflammation, and hyper-coagulation would indicate the existence of a common underlying mechanism. Thus, we hypothesized that chronic stimulation of endothelial cells (EC) with FXa would cause cell senescence, resulting in inflammation and impaired tissue regeneration.

In this study, we demonstrated that continuous FXa stimulation decreased EC proliferation, up-regulating the senescence marker such as p53, p16^INK4a^, and senescence-associated β-galactosidase (SA-β gal)-positive cells, through up-regulation of insulin-like growth factor binding protein 5 (IGFBP-5) and early growth response 1 (EGR-1). In addition, the present study demonstrated that FXa impaired angiogenesis via senescence of endothelial progenitor cell (EPC), while the direct FXa inhibitor, rivaroxaban, attenuated the impaired the angiogenic properties induced by FXa in mouse ischemic hind limb model. These data indicated that FXa induced EC and EPC senescence, leading to impaired angiogenesis.

## Results

### Chronic FXa treatment induces growth retardation and senescence of EC

**Figure 1:**
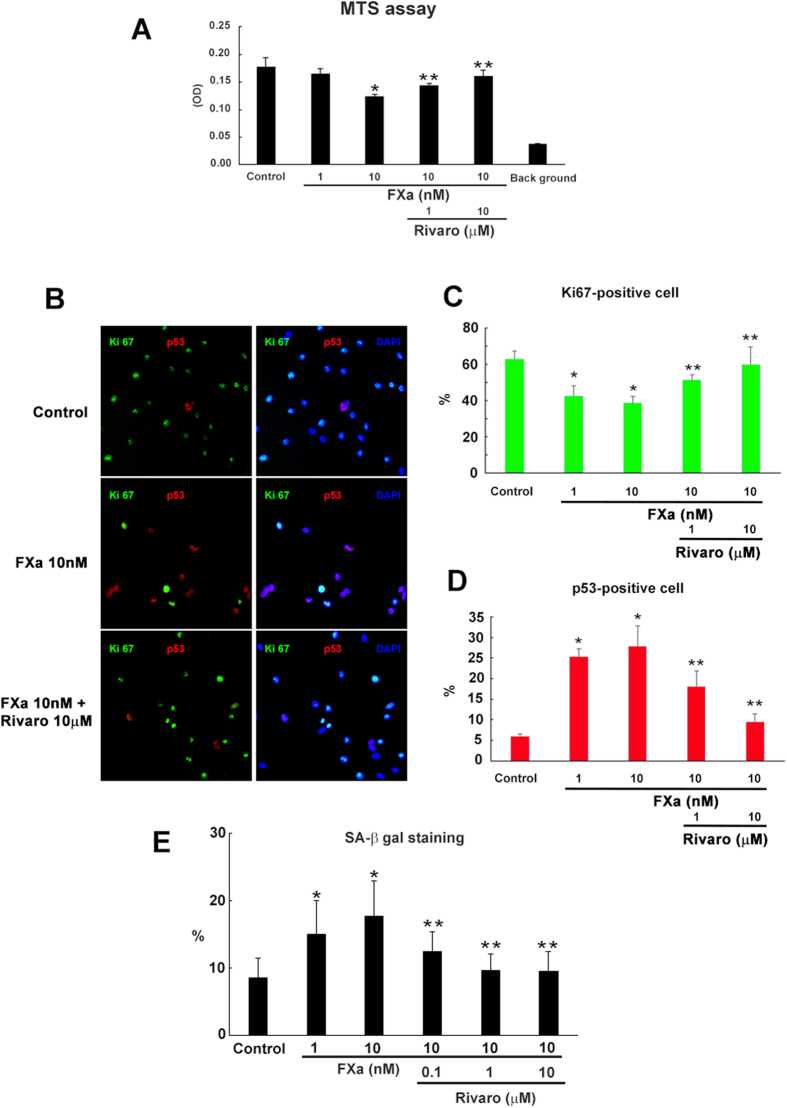
Induction of endothelial cell senescence by FXa. EC were treated every other day for 14 days with FXa (1 or 10 nM), or FXa with rivaroxaban (1 or 10 μM). Experiments were performed using samples from day 14. (**A**) EC proliferation was detected by MTS assay. ***p < 0.05 vs. control and 10 nM FXa, respectively. n = 5. (**B–D**) EC treated for 14 days were stained with Ki 67 (green), p53 (red), and DAPI (blue). Representative image of EC (**B**) and the fractions of Ki 67-positive cells (**C**) and p53-positive cells (**D**). ***p < 0.05 vs. control and 10 nM FXa, respectively. n = 3. (**E**) Senescent cells were detected by SA- β gal staining. The fractions of SA-β gal-positive ECs. ***p < 0.05 vs. control and 10 nM FXa, respectively. n = 5.

First, the effect of FXa on EC proliferation was examined. HUVECs were stimulated by FXa (1 or 10 nM) with or without rivaroxaban (10 μM) every other days for 14 days. Cell proliferation was assessed by MTS assay at day 14. Chronic FXa treatment decreased the proliferation of EC as compared to the control group in a dose-dependent manner (Fig. 1A). To further confirm the effect of FXa on EC proliferation, EC treated for 14 days with FXa were stained with the cell cycle marker, Ki67, and the cell cycle arrest marker, p53 (Fig. 1B). The fraction of Ki67-positive cells was significantly decreased with FXa treatment (Fig. 1C). In contrast, the fraction of p53-positive cells was remarkably increased with FXa treatment (Fig. 1D). Importantly, the direct inhibitor of FXa, rivaroxaban, significantly restored the proliferation of EC, and decreased the expression of p53 in EC treated with FXa.

Additionally, cell cycle analysis via FACS revealed G0/G1 arrest induced by FXa (Figure S1A and S1B). Both cell senescence and cell quiescence are associated with cell cycle arrest. Thus, SA-β gal staining was examined. As shown in Figs 1E and S1C, the fraction of SA-β gal-positive cells was significantly increased with FXa administration, and decreased by rivaroxaban co-administration. These data indicated that chronic FXa treatment inhibited the proliferation of EC at least partially through the induction of cell senescence.

### FXa-induced senescent EC increased inflammatory cytokine expression and decreased tube formation ability

**Figure 2:**
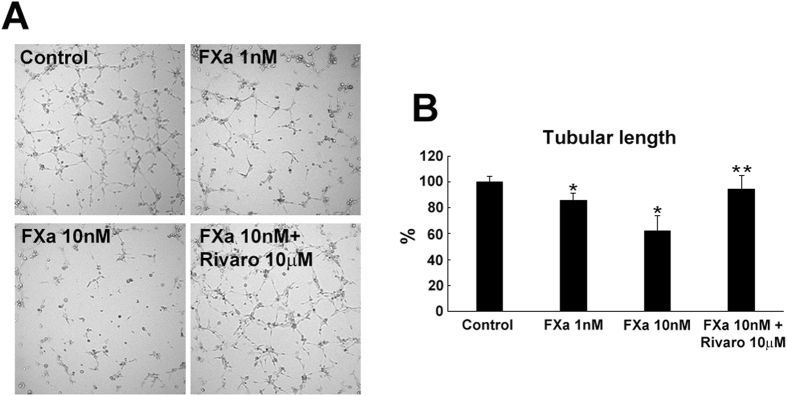
Impaired tube-formation of EC by chronic FXa treatment. Angiogenic properties were measured via tube formation assay. Representative image of Matrigel tube formation (**A**) and quantitative data (**B**). ***p < 0.05 vs. control and 10 nM FXa, respectively. n = 3.

Accumulating evidence demonstrated that senescent cells could cause the deleterious effects on the tissue microenvironment. Among these effects, the acquisition of senescence-associated secretory phenotype (SASP) that turns senescent cells into pro-inflammatory cells that have the ability to promote chronic inflammatory disease has been reported. In addition, senescent EC are functionally impaired in angiogenesis. Therefore, we compared the expression of inflammatory cytokines and the tube formation ability with FXa treatment. As shown in Figure S2A–S2D, senescent EC treated with FXa exhibited a significant increased expression of inflammatory mediators, including IL-1β, IL-6, MCP-1, and ICAM-1, while rivaroxaban significantly decreased the expression of these cytokines. Similarly, EC treated for 14 days with FXa showed impaired angiogenesis *in vitro*, as measured by tube formation assay on Matrigel, although rivaroxaban restored (Fig. 2A,B). These data strongly suggest that continuous FXa treatment induced SASP phenotype and impaired angiogenesis in EC.

### FXa induces EC senescence through IGFBP-5

**Figure 3:**
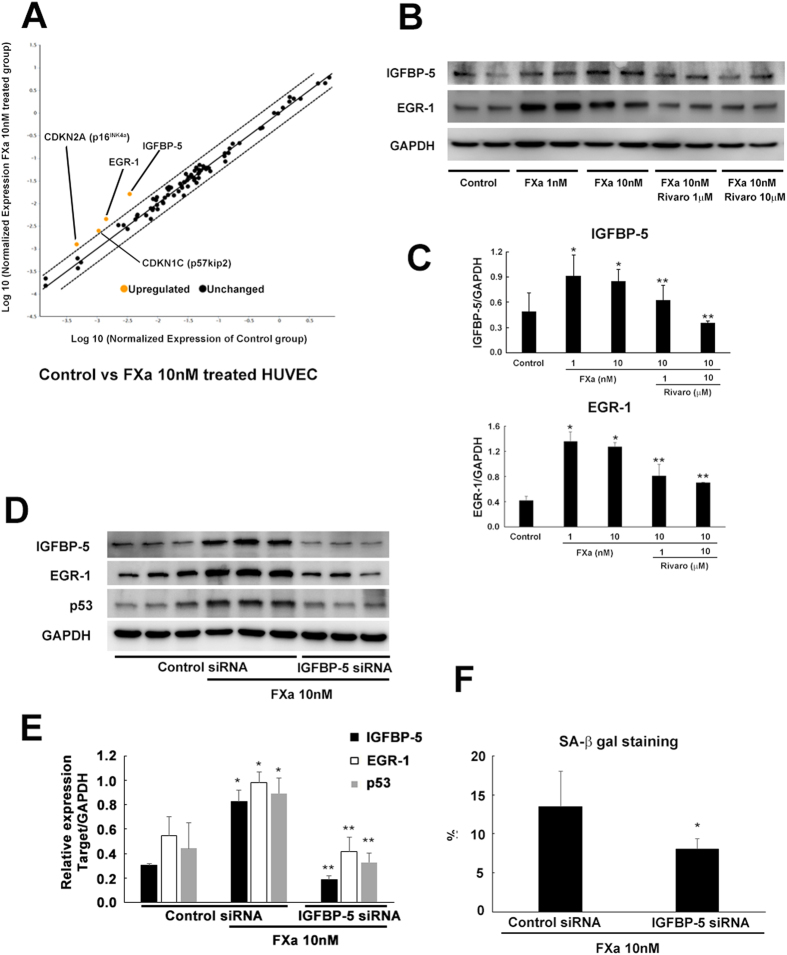
Downstream pathway of FXa-induced EC senescence. (**A**) RT2 profiler PCR array of cell senescence. Overview of scatter plot on the expression of 96 genes. Orange dots are genes upregulated in EC treated with 10 nM FXa as compared to the control group. Boundaries represent the two-fold regulation cut-off. (**B**) Expression of IGFBP-5 and EGR-1 was measured by western blotting. (**C**) Relative expression of IGFBP-5/GAPDH and EGR-1/GAPDH. ***p < 0.05 vs. control and 10 nM FXa, respectively. n = 4. (**D,E**) Expression of IGFBP-5, EGR-1, and p53 in FXa treated ECs with control siRNA or IGFBP-5 siRNA transduction were measured by western blotting. ***p < 0.05 vs. control siRNA alone and FXa + control siRNA, respectively. n = 3. (**F**) The fraction of SA-b gal-positive cells in FXa treated ECs with control siRNA or IGFBP-5 siRNA transduction. *p < 0.05 vs. control. n = 5.

Next, we focused on the mechanism how FXa induced EC senescence. Initially, we performed PCR array designed to analyze a panel of genes related to cell senescence. A scatter plot in Fig. 3A demonstrated that 4 genes including p16^INK4a^, p57^KIP2^, EGR-1, and IGFBP-5 were up-regulated in the FXa-treatment group as compared to the control group. Importantly, all 4 of these genes were down-regulated by co-administration of rivaroxaban (Figure S3A). Then, we validated the changes in the expression of these genes together with p53 by western blotting. Protein expression of IGFBP-5, EGR-1, p53, and p16^INK4a^ was significantly increased by FXa administration, and the increased expression of those proteins was almost completely abolished by rivaroxaban (Figs 3B,C, S3B, and S3C). Intriguingly, IGFBP-5 is reported to mediate both replicative and premature senescence of HUVECs through a p53-dependent mechanism *in vitro*^10^,^11^,^12^. Additionally, IGFBP-5 has been shown to induce EC senescence and lung fibrosis through EGR-1^13^. Therefore, we hypothesized that FXa induced cell senescence through IGFBP-5. Consistent with the previous report, IGFBP-5 overexpression significantly upregulated both EGR-1 and p53 (Figure S3D), and increased the fraction of SA-β gal-positive senescent EC (Figure S3E). Moreover, rhIGFBP-5 significantly increased the fraction of SA-β gal-positive senescent EC with upregulation of p53 (Figure S4A and S4B), suggesting endogenous and exogenous IGFBP-5 induce EC senescence. The ability of proliferation and tube formation was significantly impaired by IGFBP-5 overexpression (Figure S4C, S4E, and S4F). Additionally, The expression of inflammatory mediators, such as IL-1β, IL-6, and ICAM-1, were significantly upregulated by IGFBP-5 overexpression, which were not reduced by co-administration of rivaroxaban (Figure S5A–S5D). Knock down of IGFBP-5 by siRNA transduction significantly inhibited FXa-induced EGR-1 and p53 up-regulation (Fig. 3D,E) and EC senescence (Fig. 3F), together with significant inhibition of FXa-induced reduced proliferation and tube formation (Figure S4D, S4E, and S4G). Moreover, IGFBP-5 siRNA significantly down regulate FXa-induced inflammatory mediators (Figure S5E–S5H). Finally, the up-regulation of IGFBP-5 and EGR-1 by FXa was mediated through both protease-activated receptor 1 (PAR1) and PAR2 (Figure S6A–S6C). These results revealed that FXa induced EC senescence through an IGFBP-5 dependent mechanism.

### FXa-induced endothelial progenitor cell senescence impaired regeneration capacity

**Figure 4:**
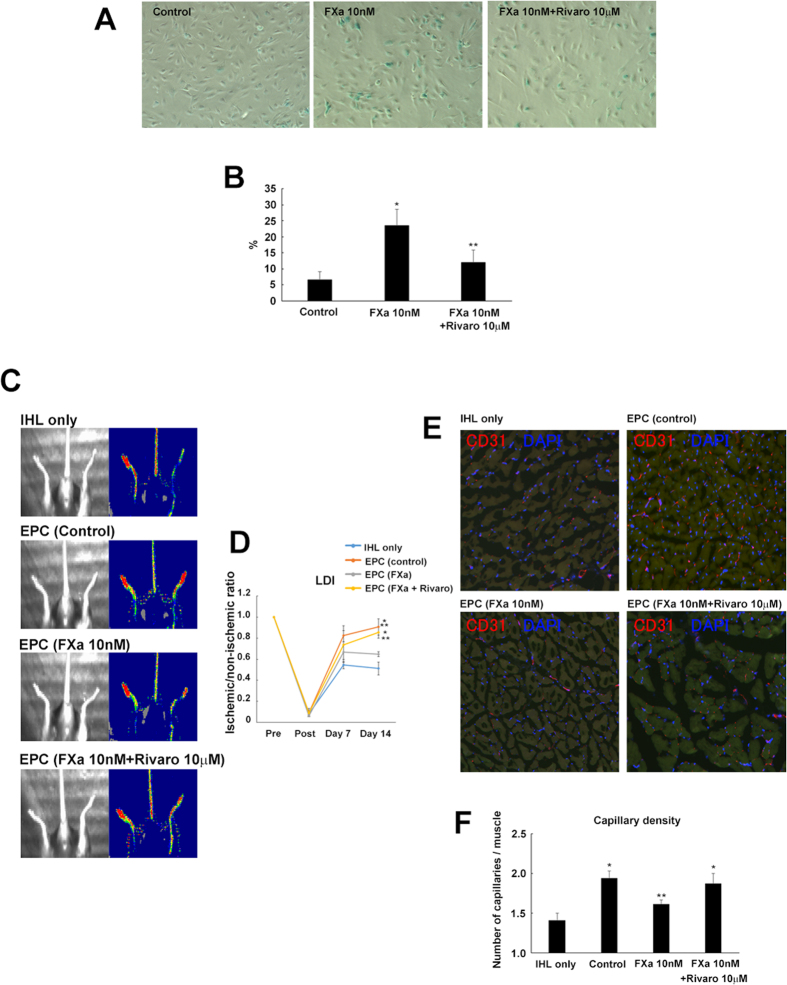
Effects of chronic FXa administration on EPC function. (**A,B**) EPC senescence was detected by SA-β gal staining. Representative image of SA-β gal staining (**A**) and the fraction of SA-β gal-positive EPC (**B**) ^***^p < 0.05 vs. control and 10 nM FXa, respectively. n = 5. (**C**) Representative image of peripheral blood flow analyzed by LDI at 14 days after femoral artery ligation. Low or no perfusion is displayed as dark blue, whereas the highest perfusion is displayed as red. (**D**) Quantitative analysis of hindlimb blood flow expressed as a ratio of ischemic hindlimb perfusion to untreated opposite limb perfusion before operation and at days 1, 7, and 14 following femoral artery ligation ^***^p < 0.05 vs. control and EPC + FXa, respectively. n = 4–5 in each group. (**E**) Representative image of capillaries in tissue sections retrieved from an ischemic leg. Adductor muscles from treated mice were stained with anti-CD31 antibodies. (**F**) Quantitative analysis of capillary density ^***^p < 0.05 vs. control and EPC + FXa, respectively. n = 4–5.

To test the hypothesis *in vivo*, endothelial progenitor cells (EPC) chronically treated with FXa were intravenously injected immediately after femoral artery ligation in a mouse ischemic hind limb model. Similar to EC, FXa significantly induced EPC senescence accompanied by IGFBP-5 and Egr-1 mRNA upregulation, which was reduced by rivaroxaban co-administration (Figs 4A,B, S7A, and S7B). Laser Doppler image (LDI) analysis showed that EPC injection enhanced the recovery of ischemic limb perfusion (Fig. 4C,D). Importantly, this beneficial effect of EPC was significantly attenuated by FXa treatment (Fig. 4C,D). Additionally, the increase in capillary density by EPC injection was also inhibited by treatment with FXa. However, co-treatment with rivaroxaban significantly increased ischemic limb perfusion and capillary density as compared to the FXa treatment group (Fig. 4E,F). Attenuation of the impaired neovascularization in rivaroxaban treatment group was accompanied by a significant increase in the number of incorporated EPCs in ischemic adductor muscle as compared to FXa treatment group (Figure S6C and S6D). These observations indicated that chronic FXa treatment induced EPC senescence and impaired EPC angiogenesis. The FXa inhibitor, rivaroxaban, may work as an anti-senescence agent and preserve the angiogenic abilities of EC and EPC.

## Discussion

Under physiological conditions, anti-coagulant system dominates the coagulation system, providing a potent defense against thrombotic complications^14^. However, in the setting of chronic inflammatory disease (e.g., atherosclerosis and inflammatory bowel disease), uncontrolled coagulation activity overwhelms the anti-coagulation system, initiating tissue inflammation and remodeling through protease-activated receptors, PARs^6^,^15^,^16^. Recent findings suggest that FXa has non-hematologic functions beyond blood coagulation that are involved in the process of atherosclerosis. In contrast, rivaroxaban, a direct FXa inhibitor, was reported to reduce the risk of recurrent atherothrombotic events in patients with acute coronary syndrome^17^,^18^. Therefore, this study aimed to evaluate the response of EC to chronic FXa stimulation, focusing specifically on cellular senescence, which is often seen in tissues affected by chronic inflammatory diseases and appears to contribute to low grade inflammation through SASP^9^.

In this study, our data for the first time demonstrated that chronic FXa stimulation caused EC senescence in an IGFBP-5-dependent manner. FXa stimulated both PAR1 and PAR2, leading to IGFBP-5-EGR-1-p53 pathway activation. Consequently, EC exhibited increased inflammatory cytokine expression and lost their angiogenic properties, resulting in impaired tissue regeneration *in vitro* and *in vivo*. FXa mediated cellular signaling via PAR1, PAR2, or both, depending on the cell type and on co-factor expression. Studies on EC and fibroblasts demonstrated that FXa stimulated secretion of inflammatory cytokines through PAR1/2^19^,^20^,^21^. In contrast, FXa mediated cell signaling mainly through PAR2 in smooth muscle cells by PAR2 mRNA stabilization. Our data consistently indicated that FXa induced IGFBP-5 expression through both PAR1 and PAR2 *in vitro*. However, active tissue factor and factor VIIa are required for FXa-mediated PAR2 signaling, and soluble FXa alone mediates PAR1 signaling. Therefore, FXa administration in “*in vitro*” experiments may raise reasonable doubt regarding “*in vivo*” relevance. Additionally, it should be noted that the presence of bovine serum in most *in vitro* study might generate thrombin from FXa in medium, which can signal through PAR1 and PAR2. Although it has not been demonstrated that thrombin induces IGFBP-5 expression, this might affect our *in vitro* observation with using PAR1 and PAR2 siRNA. Obviously, future experiments should elucidate the *in vivo* relevance of the effects of FXa on EC and EPC. Rivaroxaban itself has pleiotropic action that restore blood flow in limb ischemia. It can not only reduce thromboembolism rates in total hip or knee arthroplasty patients^22^, but also stabilize atherosclerotic plaques in a mouse model^23^. Moreover, rivaroxaban blocks the interaction between advanced glycation end products (AGEs)-RAGE axis and coagulation system to prevent thromboembolic complications in diabetes^24^, and maintains the ability of angiogenesis in diabetic mouse model^25^. Therefore, our *in vitro* study might underestimate the potential of rivaroxaban that preserves blood flow in ischemia.

By blocking FXa-mediated PAR1 and PAR2 signaling with rivaroxaban, IGFBP-5 expression, subsequent cell senescence and the production of several inflammatory mediators were significantly inhibited. Insulin/insulin-like growth factor (IGF) signaling pathways are important in the aging and longevity of many organisms, and IGFBPs tightly regulate the bioavailability of IGF^26^,^27^. Among IGFBPs, IGFBP-5 has been shown to inhibit the growth of breast cancer cells^28^, EC^10^, and fibroblasts^29^. Additionally, IGFBP-5 transgenic mice showed increased neonatal mortality and decreased whole body growth as well as decreased muscle development, indicating that excess IGFBP-5 compromises survival, growth, muscle development, and fertility in mice^30^. Our study revealed that FXa functions as an upstream effector of IGFBP-5, inducing EC and EPC senescence *in vitro*. However, it is still unclear whether FXa induces cell senescence independently of IGF as IGFBP-5 inhibits IGF signaling pathways, such as PI3K/AKT/mTOR, that affect cell apoptosis, growth, and senescence. Further studies must be performed in the future to elucidate the precise mechanism underlying FXa-induced EC senescence.

In summary, coagulation factor Xa initiated EC senescence and inflammatory cytokine production, which consequently impaired tissue regeneration. In this study, we identified IGFBP-5 as a downstream molecule of FXa-PAR signaling that activates EGR-1 and p53, leading to cell senescence. These finding may explain the observations of previous clinical trials.

## Materials and Methods

### Ethical Statement

All experimental procedures were reviewed and approved by the Institutional Animal Committee at the Department of Veterinary Science of Osaka University School of Medicine (approved number; 25-043-06) and follow the recommendations of the guidelines for animal experimentation at research institutes (Ministry of Education, Culture, Sports, Science and Technology, Japan), guidelines for animal experimentation at institutes (Ministry of Health, Labor and Welfare, Japan), and guidelines for proper conduct for animal experimentation (Science Council of Japan). Male C57BL6 mice aged 6–10 weeks were anesthetized with isoflurane for operative resection of one femoral artery, for hindlimb ischemia model. Blood flow measurement, histology, reagents, antibodies, and primers information are described in the online-only Data Supplement.

### Statistical Analysis

For statistical analysis, the values are shown as the means ± SD. ANOVA and t-tests (unpaired, 2-tailed), followed by Bonferroni adjustment for multiple comparisons were used for comparing more than two groups. A P value of < 0.05 was considered to indicate significance of mean differences.

## Additional Information

**How to cite this article**: Sanada, F. *et al.* Activated Factor X Induces Endothelial Cell Senescence Through IGFBP-5. *Sci. Rep.* **6**, 35580; doi: 10.1038/srep35580 (2016).

## Supplementary Material

Supplementary Information
