# Supplementary material for: Activated Factor X Induces Endothelial Cell Senescence Through IGFBP-5
Source: Sci Rep. 2016 Oct 18;6:35580. doi: 10.1038/srep35580 (PMC5067718; doi:10.1038/srep35580)
Supplement: Supplementary Information [file srep35580-s1.pdf]

Supplementary information for

Activated Factor X Induces Endothelial Cell Senescence Through IGFBP-5

Fumihiro Sanada[1], Yoshiaki Taniyama[1, 2], Jun Muratsu[1, 2], Rei Otsu[1],
Masaaki Iwabayashi[1], Miguel Carracedo[1], Hiromi Rakugi[2], Ryuichi Morishita[1]

1Department of Clinical Gene Therapy, 2Department of Geriatric and General Medicine, Osaka University Graduate School of Medicine, Suita, Osaka 565-0871, Japan

Running title
FXa induces endothelial cell senescence.

Address correspondence to:
Yoshiaki Taniyama, MD, PhD, Associate Professor
Department of Clinical Gene Therapy, Osaka University Graduate School of Medicine.
2-2 Yamada-oka, Suita, Osaka, 565-0871
Tel: +81-6-6210-8351, FAX: +81-6-6210-8359
E-mail address; taniyama@cgt.med.osaka-u.ac.jp
Ryuichi Morishita, MD, PhD, Professor
E-mail: morishit@cgt.med.osaka-u.ac.jp

Materials and Methods

Animal Preparation

All experimental procedures were reviewed and approved by the Institutional Animal Committee at the Department of Veterinary Science of Osaka University School of Medicine (approved number; 25-043-06) and follow the recommendations of the guidelines for animal experimentation at research institutes (Ministry of Education, Culture, Sports, Science and Technology, Japan), guidelines for animal experimentation at institutes (Ministry of Health, Labor and Welfare, Japan), and guidelines for proper conduct for animal experimentation (Science Council of Japan). C57BL6 mice aged 6-10 weeks were anesthetized with isoflurane for operative resection of one femoral artery, as described previously[1]. The ischemic/non-ischemic limb blood flow ratio was measured using laser Doppler imaging (LDI, Moor LDI-Mark 2, Moor Instruments, UK). Tissue sections from the adductor brevis muscles of ischemic limbs were harvested on day 14. The sections were stained with specific antibodies. A total of 8 different fields (in 3 cross sections from 4-5 animals) were randomly selected, and CD31-positive capillary density was determined by confocal microscopy.

Reagents and antibodies

Human FXa was purchased from BioVision Inc, California, USA. Mouse FXa was purchased from Abcam plc, Cambridge, UK. Rivaroxaban was kindly donated from Bayer pharma AG, Leverkusen, Germany. IGFBP-5 antibody was from R&D Systems, Minnesota, USA. EGR-1 antibodies were from Santa Cruz Biotechnology, Inc. Texas, USA. p53 and $p16^{INK4a}$ antibody was obtained from Cell Signaling, technology, Massachusetts, USA. Anti-mouse CD31 antibody was from BD Bioscience, California, USA. Anti-GFP antibody was from Abcam plc, Cambridge, UK. IGFBP-5 plasmid (pcDNA3-IGFBP5-V5) was purchased from addgene, Cambridge, MA, USA, and siRNA for PAR1/2 and IGFBP-5 were from Santa Cruz Biotechnology, Inc. Texas, USA. Human recombinant IGFBP-5 was from R&D Systems.

Cell culture

Human umbilical vein endothelial cells (HUVEC, passage 5 to 9) purchased from Lonza were cultured in endothelial basal medium-2 (EBM-2) (Clonetics, Walkersville, Maryland, USA) supplemented with EGM and 5 % fetal bovine serum (FBS). HUVECs were stimulated by FXa (1 or 10 nM) with or without rivaroxaban (10 μM) every other days for 14 days. Overexpression of IGFBP-5 or knockdown

experiments by siRNA was performed for 10 days in HUVEC. For endothelial progenitor cells (EPC) culture, bone marrow mononucleotide cells were isolated from C57BL/6-Tg (CAG-EGFP) mouse as described previously [2] and cultured in EBM-2 supplemented with EGM and 10% FBS. After 7 days in culture, EPCs were stimulated by FXa (1 or 10 nM) with or without rivaroxaban (10 μM) every other days for 14 days. One day after femoral artery resection, mice were received 2×10^5 culture-expanded EPC was injected intravenously.

Proliferation assay and Matrigel tubular assay,

Mitogenic activity was measured with MTS assay kit (Promega, Madison, Wisconsin, USA). We conducted Matrigel tube formation assay as described previously.[2] Briefly, 9×10^4 HUVEC were plated and incubated at 37 °C for the 24 hours on a growth factor-reduced Matrigel-coated 48 well dish. Tubular length was measured by using image J soft wear.

Analysis of senescence marker of HUVEC

Several human senescence markers of HUVEC was measured by RT^2 Profiler™ PCR Array Human Aging, QIAGEN (California, USA) as manufactures instruction. Each category consist of mixed three samples. Therefore, we validated it by RT-PCR or western botting later.

Isolation of total RNA and RT-PCR

Total RNA was isolated using RNeasy Mini Kit (QIAGEN, Hilden, Germany). DNase treated total RNA was reverse-transcribed with the High-Capacity cDNA Reverse Transcriptase Kit (Applied Biosystems, Foster City, CA, USA) to produce complementary DNA (cDNA). Reverse transcription-generated cDNA encoding the target genes was amplified and quantified by the ViiA-7™ real-time PCR system (Applied Biosystems, Foster City, CA, USA) using the primer set shown below.

Human IL-1β;
Forward TACAGTGGCAATGAGGATGAC
Reverse GTCGGAGATTCGTAGCTGGAT
Human IL-6;
Forward TGACAAACAAATTCGGTACATCCT
Reverse AGTGCCTCTTTGCTGCTTTCAC
Human MCP-1;

Forward AGTCTCTGCCGCCCTTCTGTG
Reverse TGCTGCTGGTGATTCTTCTAT
Human ICAM-1;
Forward CGACTGGACGAGAGGGATTGT
Reverse ATTATGACTGCGGCTGCTACC
Human PAR1;
Forward AGGCCAGAATCAAAAGCAACAA
Reverse CTGGTCAAATATCCGGAGGCA
Human PAR2;
Forward TGGCACCATCCAAGGAACC
Reverse CGTTACTTGGCAAACCCACC
Mouse IGFBP-5;
Forward GTGAGCCACAGATGGAGGTCATTG
Reverse TGTATCCTGGGTTCAGCCAGATC
Mouse EGR1;
Forward ACCTCTTAGGTCAGATGGAAGATC
Reverse GCAAATCAAGTCCTTTGGATAGAGG

Western Blot Analysis

Western blotting was performed as previously described.[3]

Reference

1 Sanada F. *et al*. Hepatocyte growth factor, but not vascular endothelial growth factor, attenuates angiotensin II-induced endothelial progenitor cell senescence. ***Hypertension***. **53**, 77-82 (2009).

2 Yoon CH. *et al*. Intercellular adhesion molecule-1 is upregulated in ischemic muscle, which mediates trafficking of endothelial progenitor cells. ***Arterioscler Thromb Vasc Biol***. **26**, 1066-72 (2006).

3 Sanada F. *et al*. Negative action of hepatocyte growth factor/c-Met system on angiotensin II signaling via ligand-dependent epithelial growth factor receptor degradation mechanism in vascular smooth muscle cells. ***Circ Res***. **105**, 667-75 (2009).

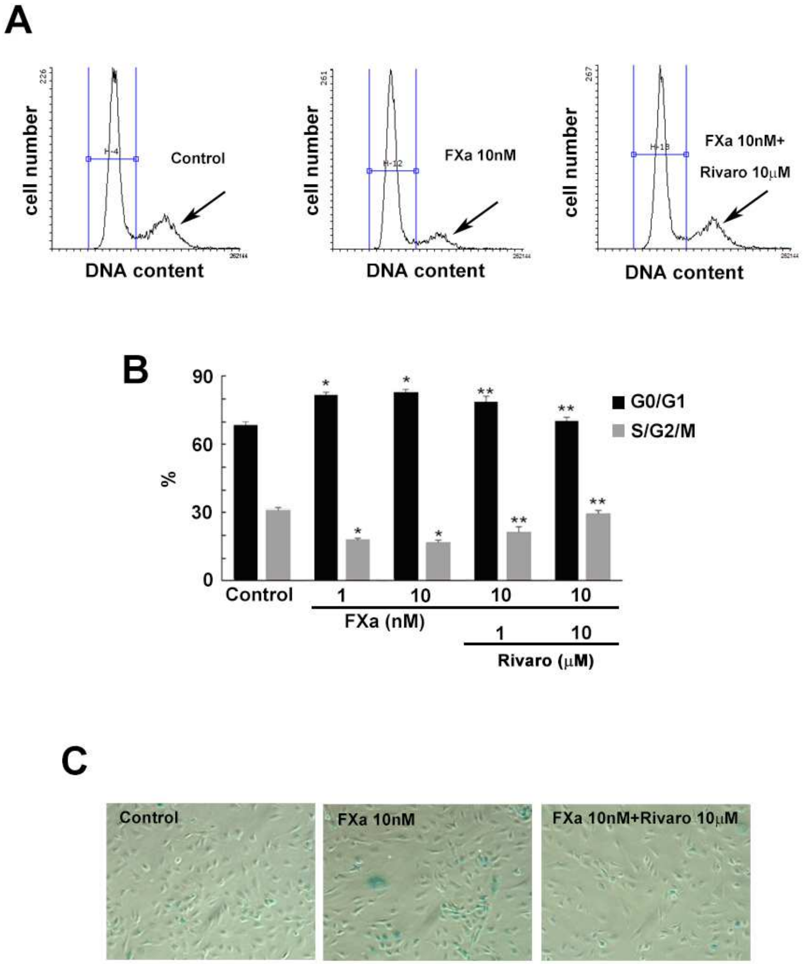

Figure S1. Induction of endothelial cell senescence by FXa.
(A and B) Cell cycle distribution. Cell cycle distributions were measured by flow cytometry using DNA content (A). Respective proportions of G0/G1 and S/G2/M (B). *, **$p<0.05$ vs. control and 10 nM FXa, respectively. n=4. (C). Senescent cells were detected by SA-β gal staining. Representative image of SA-β gal staining. n=5.

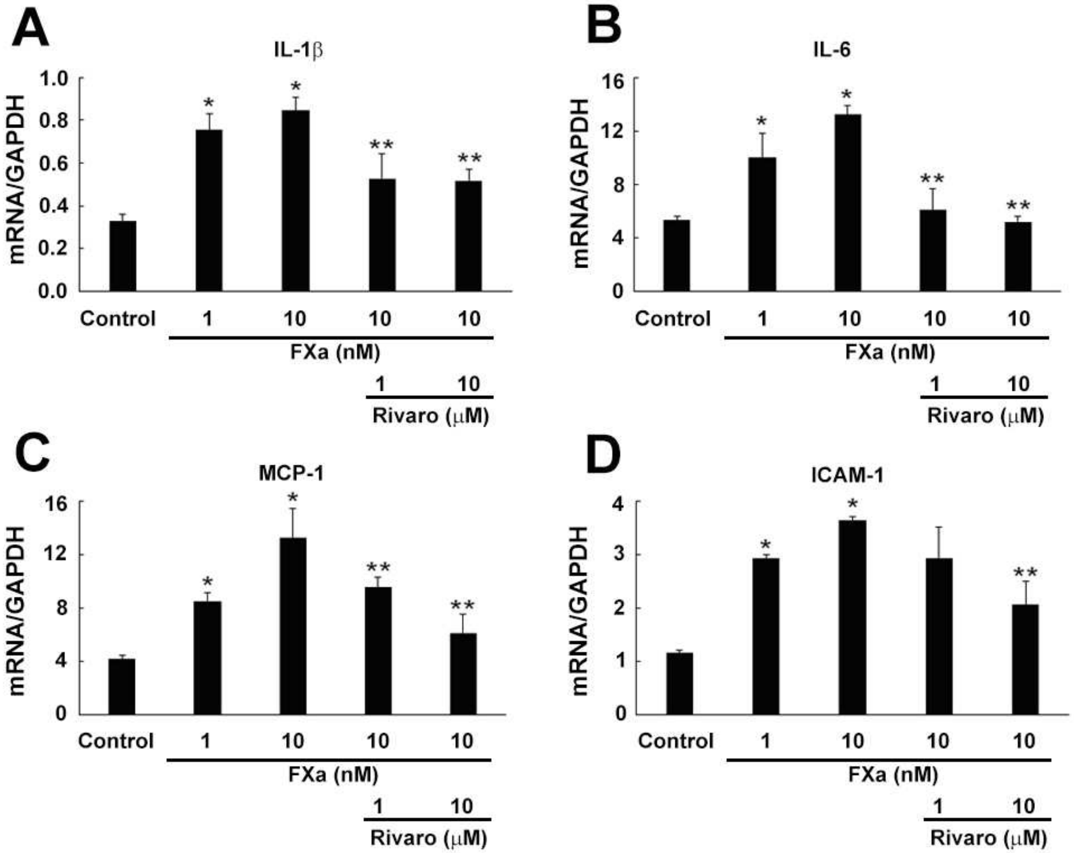

Figure S2. Induction of inflammatory mediators by chronic FXa treatment.
Several inflammatory cytokines were measured by quantitative RT-PCR, including IL-1β (A), IL-6 (B), MCP-1 (C), and ICAM-1 (D). *, **$p<0.05$ vs. control and 10 nM FXa, respectively. n=3.

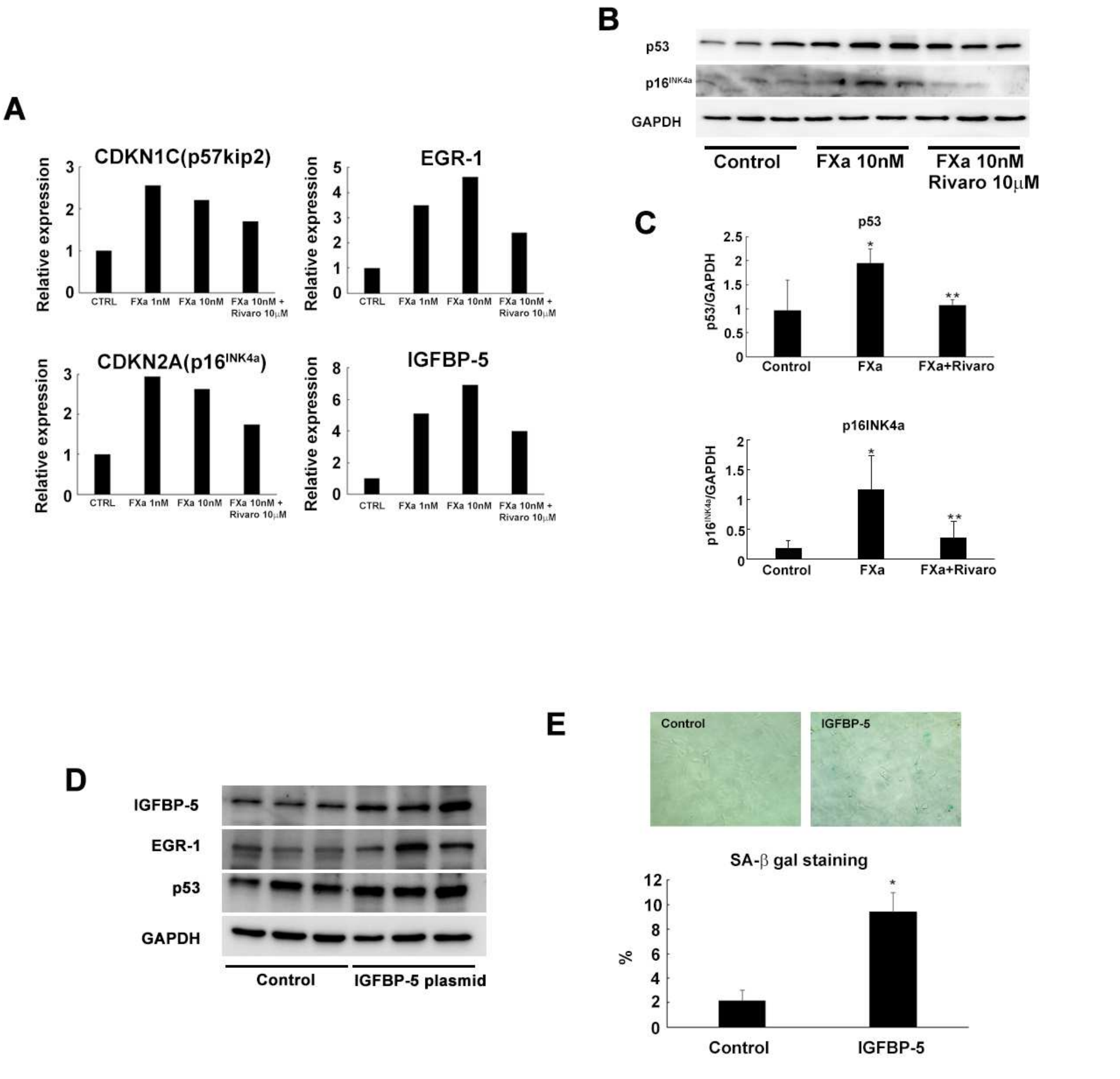

Figure S3. Downstream pathway of FXa-induced EC senescence.
(A) RT2 profiler PCR array of cell senescence. Four genes up-regulated in EC treated with 10 nM FXa as compared to the control group. (B) Expression of p53 and $p16^{INK4a}$ was measured by western blotting. (C) Relative expression of p53/GAPDH and $p16^{INK4a}$/GAPDH. *, **$p<0.05$ vs. control and FXa, respectively. n=3. (D) Expression of IGFBP-5, EGR-1, and p53 followed by IGFBP-5 over-expression was measured by western blotting. (E) IGFBP-5 induced ECs senescence was measured by SA-β gal staining. Representative image of SA-β gal staining (upper panel) and the fraction of SA-β gal-positive ECs was shown in lower graph. *$p<0.05$ vs. control. n=7.

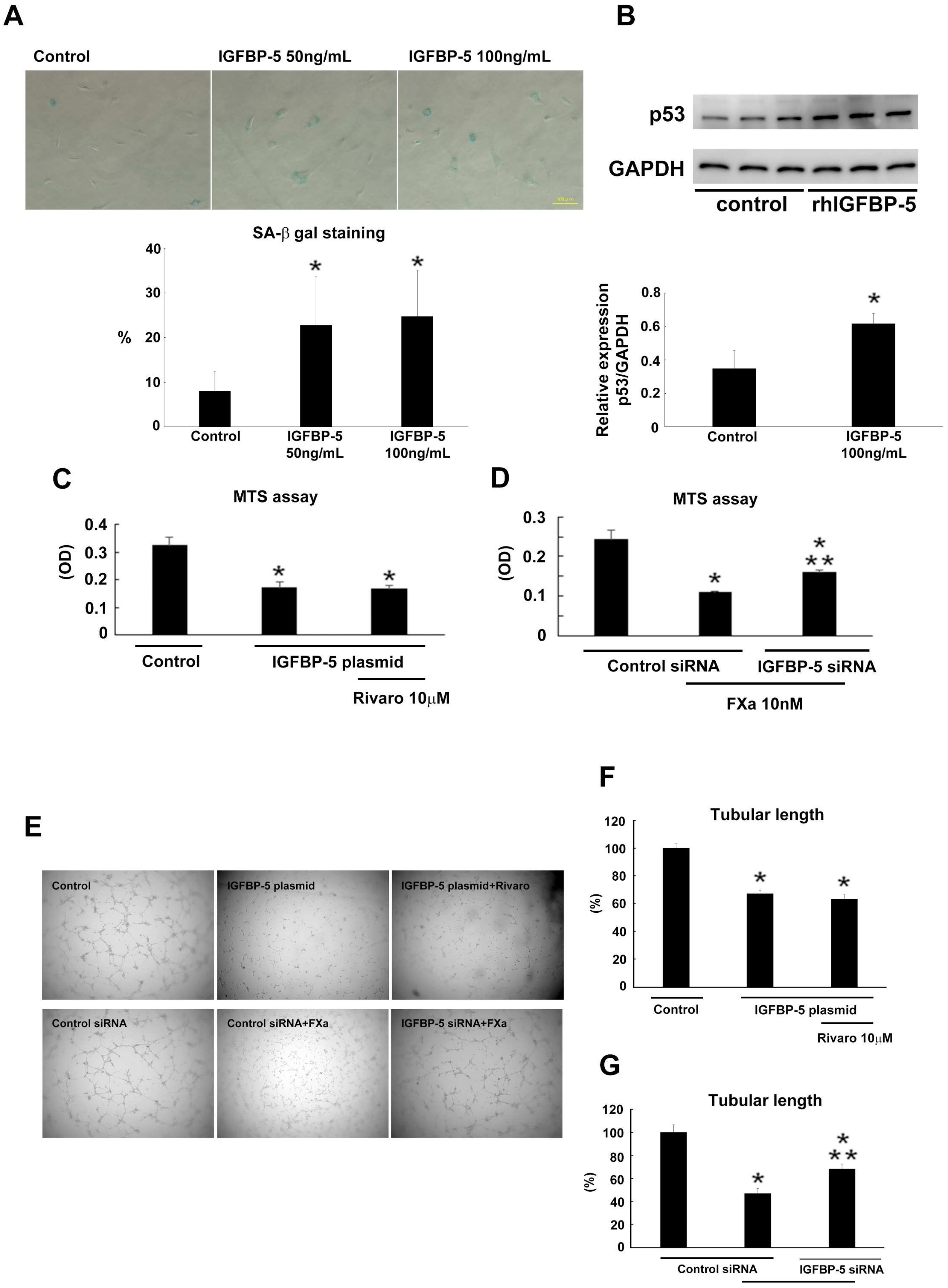

Figure S4. FXa induces ECs senescence through IGFBP-5
(A and B) rhIGFBP-5 increases EC senescence. HUVECs were incubated with rhIGFBP5 for 48hrs. (A) SA-β gal staining, n=5, *P<0.01 vs. Control. (B) p53 expression, n=3, *P<0.01 vs. Control. (C and D) IGFBP-5 overexpression decreases proliferation (C) and IGFBP-5 siRNA restores FXa-induced impaired proliferation (D). n=3, *,**P<0.01 vs. Control and control siRNA+FXa 10nM, respectively. (E-G) Tube formation assay. Representative images (E) and quantification of tubular length(F and G). n=3, *,**P<0.01 vs. Control and control siRNA+FXa 10nM, respectively.

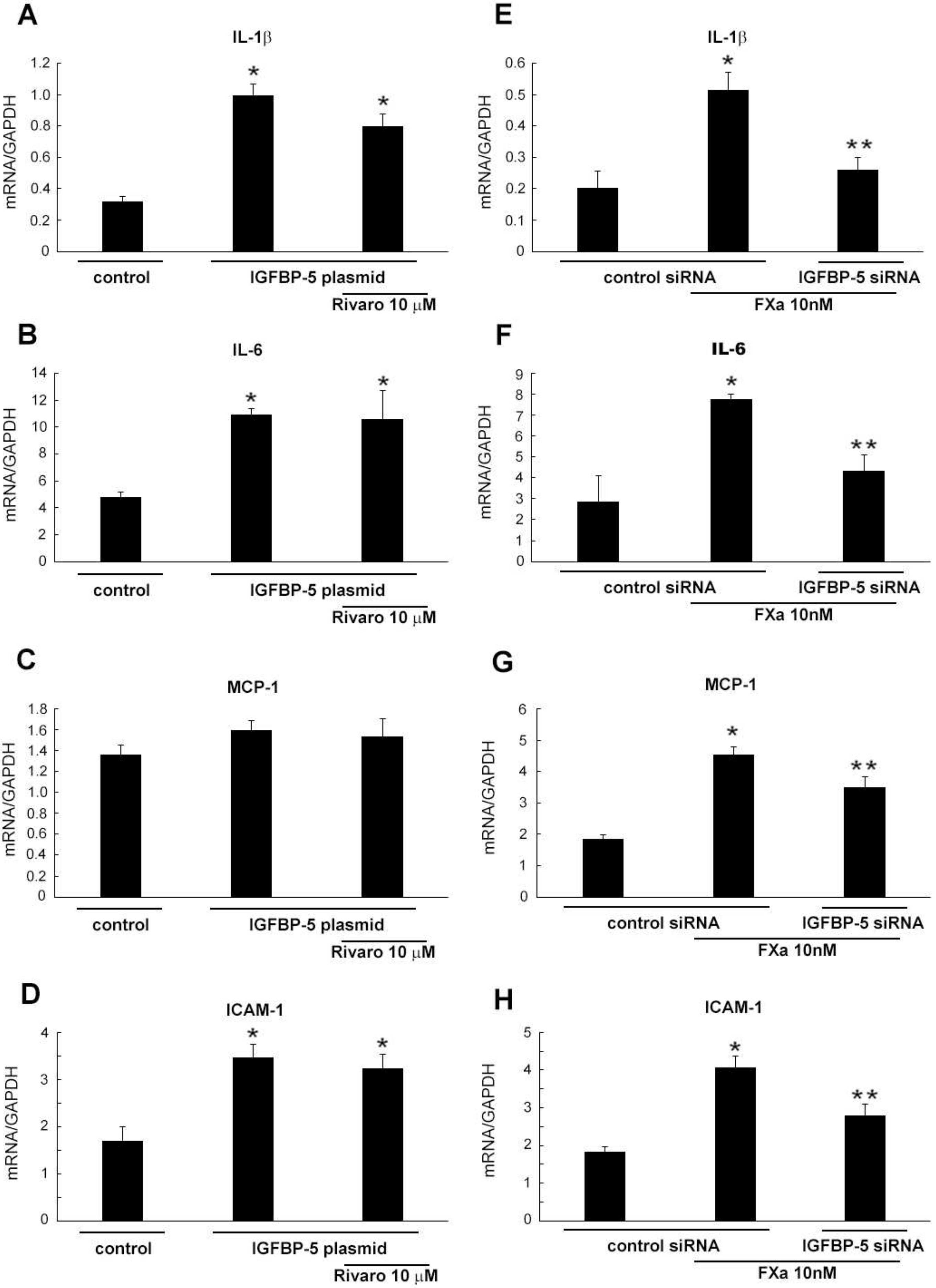

Figuar S5. IGFBP-5 induced senescent cells increases inflammatory mediators expression.

IL-1β, IL-6, MCP-1, and ICAM-1 expression following IGFBP-5 overexpression (A-D) and IGFBP-5 knock down (E-H).

n=4, *,**P<0.01 vs. control and control siRNA+FXa 10nM, respectively.

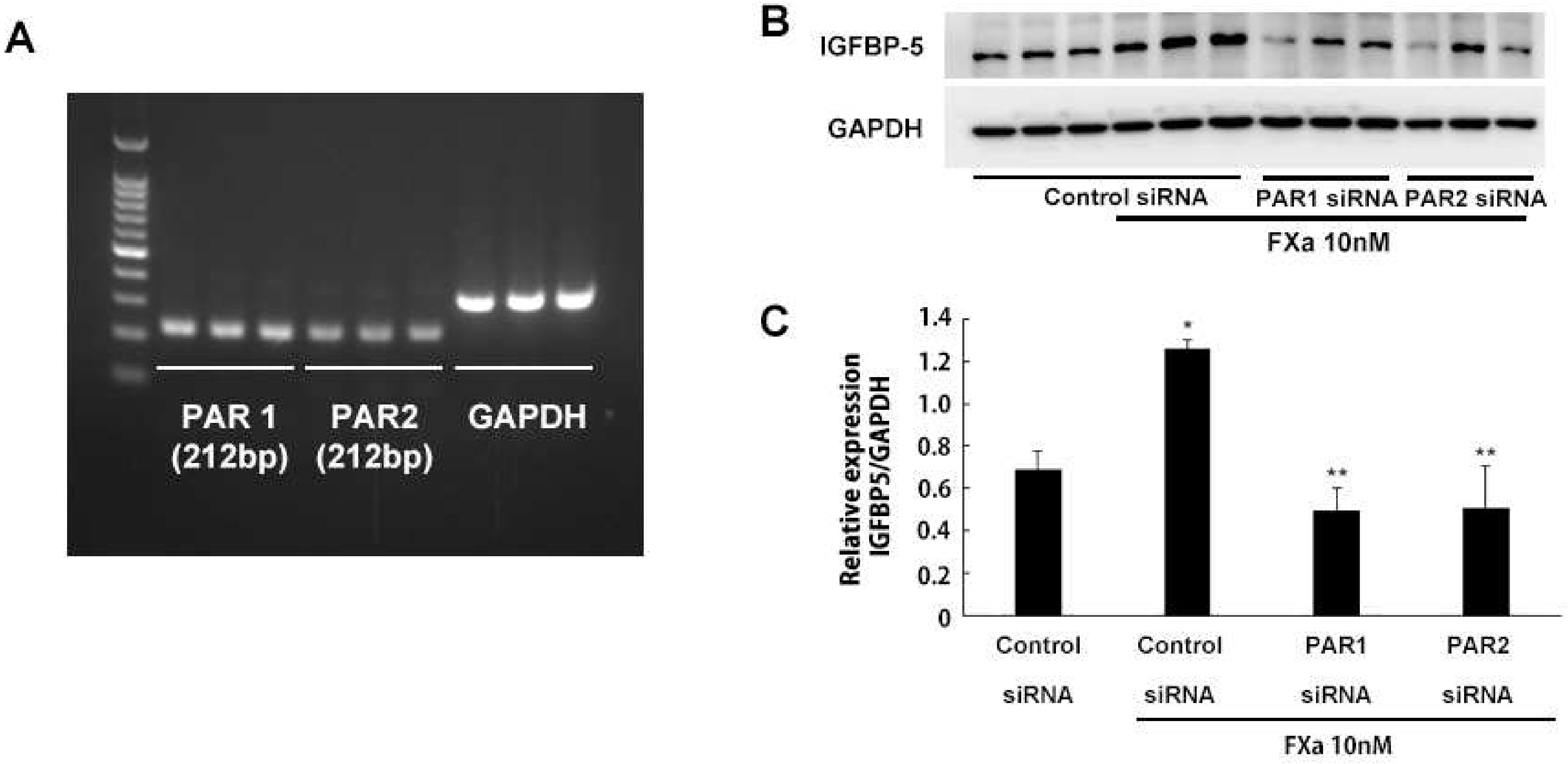

Figure S6. FXa-induced IGFBP-5 expression through PAR1 and PAR2

(A)PAR1 and PAR2 mRNA expression in HUVEC.

(B and C) Expression level of IGFBP-5 in FXa treated EC with control, PAR1 and PAR2 siRNA transduction. *, **$p<0.05$ vs. control siRNA alone and control siRNA+FXa, respectively. n=3.

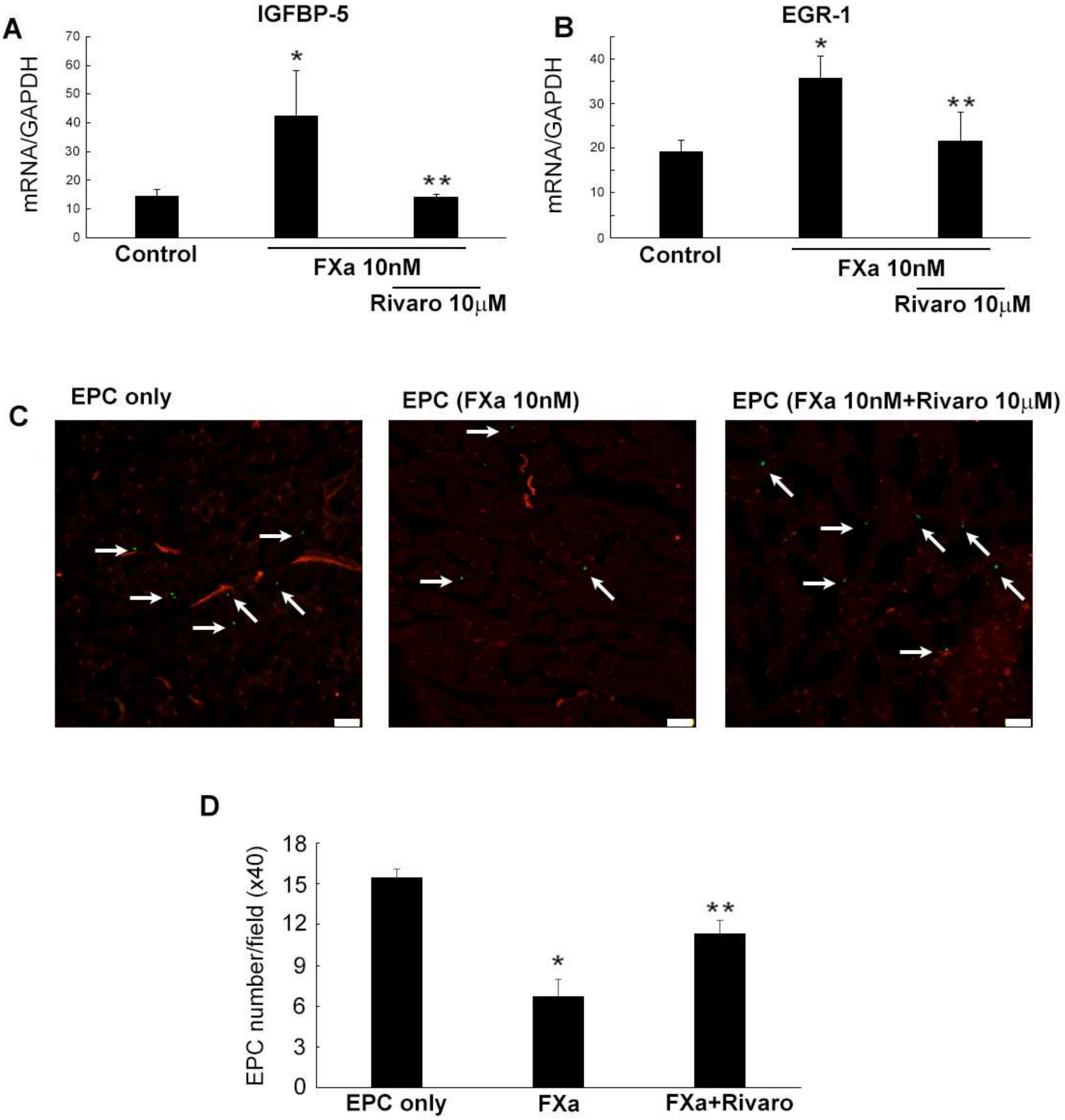

Figure S7. FXa-induced EPC senescence.

(A and B) IGFBP-5 (A) and EGR-1 (B) mRNA expression following FXa stimulation in EPC. *, **p<0.05 vs. control and FXa, respectively. n=4. (C and D) Incorporation of injected EPC into ischemic leg.(C) Representative image of double fluorescence staining in ischemic limb on day 14. CAG-EGFP mice derived EPC are identified as green and CD31-positive cell are Red. Scale bars indicate 50 μm. (D) Quantitative analysis of EPC and host CD31-positive endothelial cells in tissue sections of ischemic adductor muscles. n=4-5, *,**P<0.01 vs. EPC only and FXa, respectively.
